# The CAF-1 and Hir Histone Chaperones Associate with Sites of Meiotic Double-Strand Breaks in Budding Yeast

**DOI:** 10.1371/journal.pone.0125965

**Published:** 2015-05-04

**Authors:** Elsa Brachet, Claire Béneut, Maria-Elisabetta Serrentino, Valérie Borde

**Affiliations:** 1 Institut Curie, Centre de Recherche, Paris, France; 2 CNRS, UMR 3664, Paris, France; Tulane University Health Sciences Center, UNITED STATES

## Abstract

In the meiotic prophase, programmed DNA double-strand breaks (DSB) are introduced along chromosomes to promote homolog pairing and recombination. Although meiotic DSBs usually occur in nucleosome-depleted, accessible regions of chromatin, their repair by homologous recombination takes place in a nucleosomal environment. Nucleosomes may represent an obstacle for the recombination machinery and their timely eviction and reincorporation into chromatin may influence the outcome of recombination, for instance by stabilizing recombination intermediates. Here we show in budding yeast that nucleosomes flanking a meiotic DSB are transiently lost during recombination, and that specific histone H3 chaperones, CAF-1 and Hir, are mobilized at meiotic DSBs. However, the absence of these chaperones has no effect on meiotic recombination, suggesting that timely histone reincorporation following their eviction has no influence on the recombination outcome, or that redundant pathways are activated. This study is the first example of the involvement of histone H3 chaperones at naturally occurring, developmentally programmed DNA double-strand breaks.

## Introduction

During meiosis, programmed DSB formation and their subsequent repair as crossovers ensure the accurate segregation of homologous chromosomes and the formation of gametes with a normal chromosomes content. DSBs are catalyzed by the Spo11 transesterase, homolog of the A-subunit of Topo VI, an archaeal type II topoisomerase. After DSB catalysis, Spo11 is removed by endonucleolytic cleavage to allow 5’ ends resection and the production of 3’ tails. These 3’ tails invade a homologous intact duplex to initiate repair by homologous recombination [1]. Genetic and physical evidences have shown that meiotic DSBs are repaired via two major pathways, leading to either noncrossover (NCO) or crossover (CO) products [2, 3]. The vast majority of NCO events arise via re-annealing of the invading strand with the parental duplex after DNA synthesis using the intact template (synthesis-dependent strand annealing or SDSA) [4, 5]. COs arise mainly via the stabilization of early recombination intermediates, leading to the formation of double Holliday junctions (dHJ), that are subsequently asymmetrically resolved, presumably by the MutLγ mismatch repair Mlh1-Mlh3 heterodimer [2, 6]. This crossover pathway is promoted through the action of a group of proteins collectively named “ZMM”, whereas SDSA is favored by anti-crossover helicases, such as Sgs1 in budding yeast [7] and FANCM in plants and fission yeast [8–10].

A current model for the control of CO formation is that ZMM proteins act as chaperones to protect early dHJ recombination intermediates precursors from dismantling by the anti-crossover helicases [11]. Strikingly, in the absence of Sgs1, intermediates of recombination lead to joint molecules that are less sensitive to the ZMM and Mlh1-Mlh3 pathway, but instead depend on structure-specific nucleases, Mus81, Slx4 and Yen1, for their resolution [6, 7]. Resolution of these joint molecules produces both CO and NCO products, similar to what occurs upon repair by homologous recombination in somatic cells. In wild-type cells, about 10% of all COs (depending on the loci) arise through this pathway, meaning that some recombination intermediates escape the action of Sgs1 and thus fail to go to the ZMM pathway.

ZMM-dependent crossovers (also called class I) show a non-random distribution, such that CO tend to be widely and evenly spaced (a phenomenon called interference), whereas COs produced by the alternative pathway (class II) are not [12–14].

In budding yeast, DSB are formed within the short nucleosome-depleted regions (NDR) of ca. 140 bp in gene promoters [15]. However, their repair by homologous recombination involves sequences flanking the breaks that are within a nucleosome context (e.g. [15]). Nucleosomes may represent an obstacle for the recombination process (reviewed in [16]). In somatic cells, nucleosomes flanking a site-specific induced DSB are evicted, at least at a few hundred bp from the break, which represents the eviction of one or two nucleosomes [17–20]. Nucleosomes are then reincorporated upon break repair, both to restore the evicted nucleosomes and to erase repair-specific histone modifications [17, 19, 21].

There are several evidences that chromatin plays a role during meiotic recombination. Specific histone modifications or variants are involved: H3K4 methylation is important in budding yeast [22], H3K9 acetylation is important in fission yeast [23] and H2AZ is important in the plant *Arabidopsis* [24].

Specific factors called histone chaperones have the ability to incorporate nucleosomes into chromatin [25]. In mammalian cells, the conserved CAF-1 histone H3 chaperone is recruited to DNA lesions and promotes histone reincorporation coupled to DNA synthesis at UV lesions, as it does during DNA replication [26, 27]. CAF-1 is recruited to both UV lesions (repaired by NER) [28, 29] and to laser-induced DSBs (repaired by NHEJ or homologous recombination) [30]. Both types of DNA lesions experience DNA synthesis upon their repair, and CAF-1 complex recruitment to UV lesions is dependent on its interaction with PCNA [31]. CAF-1 is composed of three subunits, called Cac1, Cac2 and Cac3 in budding yeast [32].

Another histone H3 chaperone, HIRA (Hir in budding yeast), promotes nucleosome assembly independently of DNA replication [33]. In mammalian cells, HIRA is specific for the non-replicative H3 variant, H3.3. Its function consists mainly to reassemble nucleosomes at gaps left by transcription [34]. HIRA was recently described to be recruited to DNA lesions, both at laser-induced DSB lesions [35] and to UV lesions [36]. In the latter case, HIRA recruitment depends on the CUL4A-DDB1 complex and ubiquitylation, and is important for transcription restart after repair of the UV lesions [36]. Its mode of recruitment to DSBs is currently unknown.

Although in wild-type cells CAF-1 and HIRA mediate DNA synthesis-dependent and—independent nucleosome assembly, respectively, in many instances HIRA can compensate for the absence of CAF-1. An indication of their functional redundancy comes from budding yeast, where double mutants of the Hir and CAF-1 complexes show synthetic growth defects [37]. Several phenotypes, such as failure to deactivate the checkpoint following DNA damage, are seen only when the two histone H3 deposition modes are inactivated [21]. In mammalian cells, HIRA is able to take over for nucleosome assembly on newly replicated DNA in the absence of CAF-1 [34].

In the context of meiotic recombination, the DNA synthesis step during which replication-coupled chromatin reassembly may occur leads to either double Holliday junction formation (CO pathway) or dismantling of invasion intermediates and repair by SDSA (NCO pathway) [2]. Since recombination intermediates in meiosis are subjected to the combined actions of pro- or anti-crossover activities, we thought that chromatin reassembly during recombination intermediates formation may be an additional layer regulating the fate of meiotic recombination. We thus decided to study if nucleosomes were mobilized around meiotic DSBs, if histone chaperone activities were at play at the DSB sites, and finally if their action may have an impact on the meiotic recombination outcome.

## Results

### Histone loss occurs transiently at a site-specific meiotic DSB

**Fig 1:**
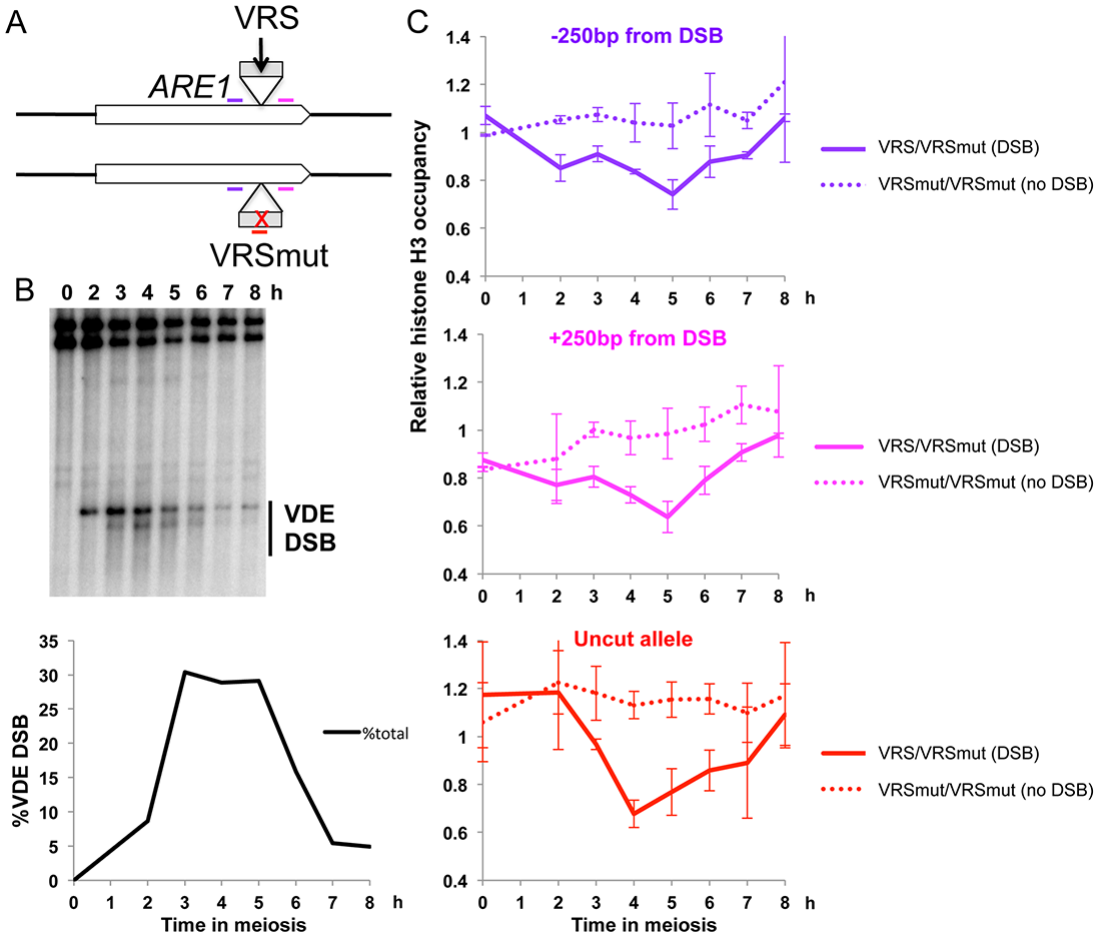
Nucleosomes are disrupted in response to meiotic DSB. (A) Experimental system. One homolog carries the VDE recognition site (VRS) and the other, VRS mut. VRS is inserted in the *ARE1* gene on chromosome III, 1.4 kb after ATG. DSB at VRS are formed during meiosis and repaired by gene conversion with the VRS mut allele. The regions amplified by qPCR are indicated by a purple (250bp 5’ of the VDE site), pink (250bp 3’ of the VDE site) or red (VRS mut allele) bar. (B) VDE DSB formation upon time course of VDB1386 monitored by Southern blot. DNA at the indicated times was digested with *Bgl*II restriction enzyme. (C) Relative histone H3 occupancy in the proximity of the break, using qPCR primers placed 250bp away on each side of the VDE break or in the mutated VRS sequence (uncut allele). ChIP of histone H3 from VBD1386 (VRS::*ARE1*/VRS mut::*ARE1*) and from VBD1389 (VRS mut::*ARE1/*VRS mut::*ARE1*) meiotic time course. Error bars represent standard deviation from two independent ChIP experiments. Histone occupancy is measured relative to histone occupancy at 9.9 kb 3’ from the VDE break.

To investigate the potential role of histone loss and replacement on meiotic recombination, we first asked if histone loss occurs at a DSB formed and repaired in meiotic cells. To maximize our chance of detecting such events, we chose to study a DSB that is formed in 100% of the cells. This represents five times more than the most active natural Spo11 hotspot (50% versus 10% of the chromatids are cut) [15]. Such DSB is induced naturally by the site-specific VDE endonuclease during the meiotic prophase, at the same time as Spo11-induced DSB [38]. We introduced a VDE recognition sequence (VRS) within the *ARE1* gene (at +1.4 kb/ATG) on chromosome III on one homolog, and on the other homolog, we inserted at the same allelic position the same sequence mutated for four nucleotides (VRS mut), resulting in an absence of VDE cleavage [39] (Fig 1A). This heteroallelic system allows repair of the DSB with the uncleaved homolog, and monitoring nucleosome loss on both alleles or only on the donor (uncleaved) molecule, thanks to specific PCR primers (Fig 1A).

During a meiotic time-course, the VDE site inserted in *ARE1* was cleaved from two hours after the meiotic onset and was repaired by seven hours (Fig 1B). When investigated in parallel, we observed a loss of histone H3 at 250 bp each side from the break, which was not seen in the control strain containing both VRS mut alleles (Fig 1C). Histone loss reached a maximum at 5 hours in meiosis, after which histone H3 reached back its initial level. Interestingly, histone H3 was also lost from the donor, uncleaved allele, only if the VRS was cleaved on the homolog, likely reflecting histone loss occurring upon strand invasion by the broken DNA and joint molecule formation, similar to what was observed at a site-specific DSB in vegetative yeast cells [19] (Fig 1C, lower panel).

We could not detect histone H3 loss around two natural Spo11 DSB hotspots, *ERG1* on chromosome VII and *GAT1* on chromosome VI (data not shown). However, since these DSB are formed in 10% of the chromatids, we consider it unlikely that we would have been able to detect histone loss due to the sensitivity of our system.

We conclude that histones are transiently lost surrounding a DSB formed in meiotic cells, both on the cleaved and uncleaved alleles.

### The CAF-1 histone chaperone is recruited to meiotic DSB independently of strand invasion and of its interaction with PCNA

We next investigated if the CAF-1 histone chaperone associates with sites of meiotic recombination. Since we detected a histone loss at the VDE break, we monitored association of tagged Cac1 (Cac1-3HA), the large CAF-1 subunit, by chromatin immunoprecipitation both with the VDE and with natural Spo11 sites during meiosis.

**Fig 2:**
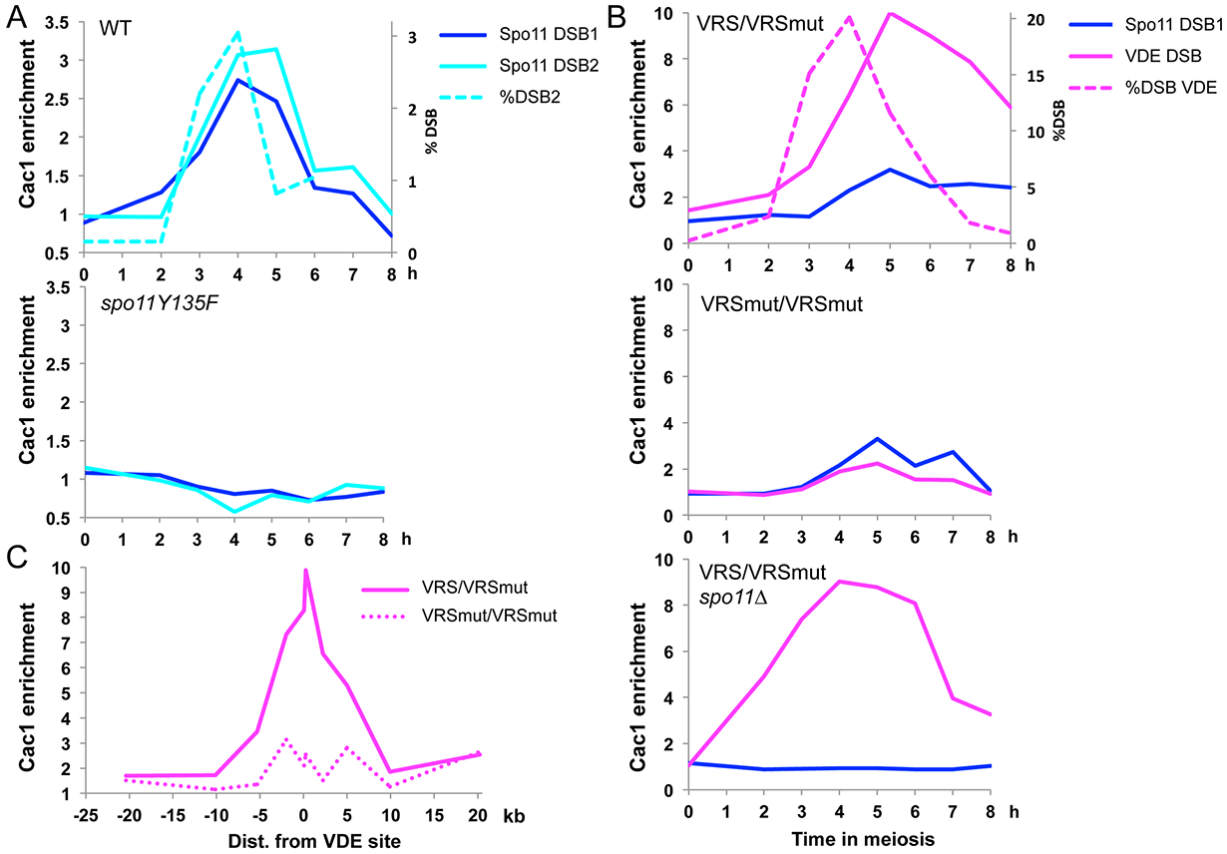
The CAF-1 large subunit is recruited to Spo11 and VDE meiotic DSBs. (A) Cac1-3HA association with two Spo11 DSB sites, DSB1 (*GAT1* promoter) and DSB2 (*BUD23* promoter). Enrichment at each site is measured relative to that at a negative control site. ChIP from WT (VBD1098) and from *spo11Y135F* (VBD1101) meiotic time course at the indicated times in meiosis. DSB formation during VBD1098 time course was monitored at the *BUD23* promoter by Southern Blot and quantified (upper panel). (B) Cac1-3HA association with the VDE DSB site inserted at *ARE1* and with one Spo11 DSB site (*GAT1* promoter). ChIP from VBD1115 (VRS::*ARE1/*VRS mut::*ARE1*), VBD1161 (VRS mut::*ARE1*/VRS mut::*ARE1*) and VBD1211 (VRS::*ARE1*/VRS mut::*ARE1 spo11Δ*) meiotic time courses. VDE DSB formation during VBD1115 time course was monitored by Southern blot and quantified. (C) Cac1-3HA association around the VDE DSB site, using qPCR primers located at different distances from the VDE break. ChIP samples are from a +4h time-point from VBD1115 (VRS::*ARE1/*VRS mut::*ARE1*) and from VBD1161 (VRS mut::*ARE1/*VRS mut::*ARE1*) meiotic time courses.

Cac1 associated with two Spo11 DSB hotspots, *GAT1* (DSB1) and *BUD23* (DSB2), at the time of DSB formation, and in a way that depends on Spo11 cleavage (Fig 2A). Similarly, Cac1 associated with the VRS DSB site, and not in a control strain bearing both VRS mut alleles (Fig 2B). Cac1 binding was specific to the VDE DSB, because it occurred also in *spo11Δ* cells (Fig 2B, lower panel). Comparison between VRS/VRS mut and VRS mut/VRS mut strains allowed us to estimate the size of the DNA region bound by Cac1 upon VDE DSB formation to 5 to 10 kb each side of the break site (Fig 2C).

**Fig 3:**
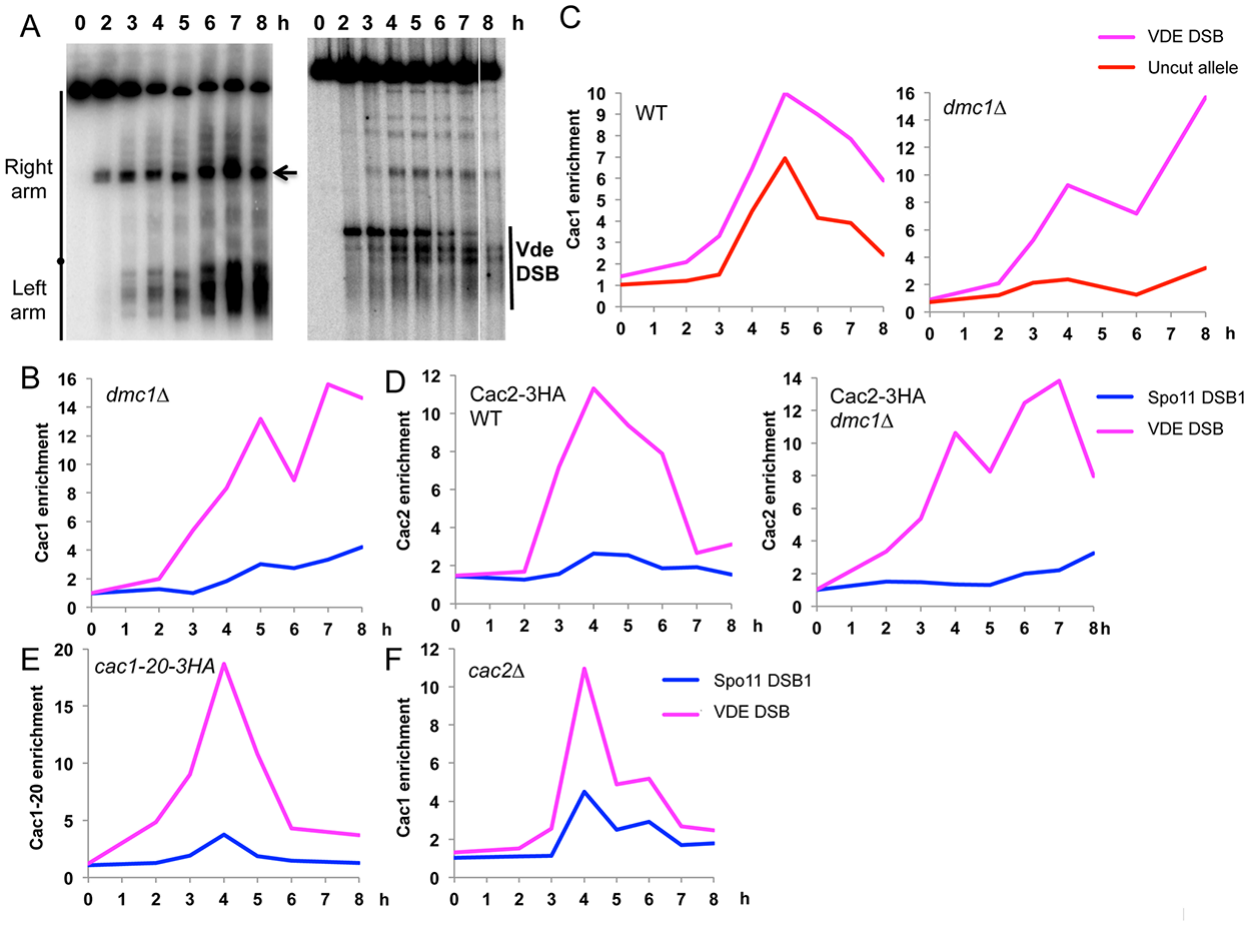
CAF-1 large subunit is recruited to Spo11 and VDE meiotic DSB, independently of strand invasion and of its interaction with PCNA. (A) VDE DSB formation upon a time course of VDB1206 (VRS::*ARE1/*VRS mut::*ARE1 dmc1Δ)* monitored by Pulse-Field Gel Electrophoresis of whole chromosomes followed by Southern blot and hybridization with a *CHA1* probe at the left end of chromosome III (left panel) or by regular Southern blot after restriction digest with *Bgl*II (right panel). (B) Cac1-3HA association with the VDE DSB site and a Spo11 DSB site (*GAT1* promoter) relative to the negative control site in a *dmc1Δ* strain. ChIP is from a VBD1206 meiotic time course. (C) Cac1 is recruited to the donor DNA (uncut allele) in wild-type but not in *dmc1Δ*. Cac1-3HA association with VDE DSB site and the uncut VRS site relative to the control site. ChIP from VBD1115 (VRS::*ARE1/*VRS mut::*ARE1*) and VBD1206 (VRS::*ARE1/*VRS mut::*ARE1 dmc1Δ*) meiotic time course. (D) Cac2 is recruited to the VDE and Spo11 DSBs, in wild-type and *dmc1Δ*. ChIP from VBD1331 (VRS::*ARE1*/VRS mut::*ARE1*) and from VBD1349 (*dmc1Δ* VRS::*ARE1*/VRS mut::*ARE1*) meiotic time courses. (E) Cac1 is recruited to VDE and Spo11 DSBs, independently of its interaction with PCNA. ChIP from a VBD1314 (VRS::*ARE1*/VRS mut::*ARE1 cac1-20-3HA*) meiotic time-course. (F) Cac1 is recruited to VDE and Spo11 DSBs, independently of the Cac2 subunit. ChIP from VBD1281 (VRS::*ARE1*/VRS mut::*ARE1 cac2Δ*) from a meiotic time course.

CAF-1 promotes nucleosome assembly coupled to DNA replication, and thus its association to sites of recombination would be expected to occur during the DNA synthesis step of homologous recombination, after strand invasion. To investigate when CAF-1 is recruited to meiotic DSBs, we used *dmc1Δ* strains, where recombination stops before strand invasion and resected DSB ends accumulate (Fig 3A). To our surprise, Cac1 associated with DSBs and accumulated during the time-course (Fig 3B). Importantly, although Cac1 association on the donor DNA (uncleaved allele) was detected in wild-type (Fig 3C, left panel), it did not occur in *dmc1Δ*, consistent with the fact that there was no strand invasion at the VDE break (Fig 3C, right panel). Similarly, the Cac2 subunit was also recruited to the VDE DSB, both in repair-proficient and in *dmc1Δ* cells, suggesting that the whole CAF-1 complex is recruited to DSBs (Fig 3D).

These results raise the question of the factors involved in CAF-1 recruitment to meiotic DSBs. For replication-coupled nucleosome assembly during S phase and UV repair in mammalian cells, CAF-1 is recruited to DNA through its interaction with PCNA [31]. We investigated the recruitment of Cac1 mutated for its interaction with PCNA (*cac1-20* mutant, [40]) to meiotic DSB. Surprisingly, the PCNA interaction-defective mutant Cac1 was recruited to DSBs (Fig 3E), indicating that association with PCNA is not needed to target Cac1 to DSBs. Finally, it is noteworthy that Cac1 was recruited to DSB even in the absence of the Cac2 subunit, suggesting that Cac1 may be the target of the recruiting event (Fig 3E).

Altogether, our results show that the CAF-1 complex is recruited to meiotic DSBs, prior to strand invasion and independently of its interaction with PCNA.

### The histone chaperone Hir is recruited to meiotic DSBs

**Fig 4:**
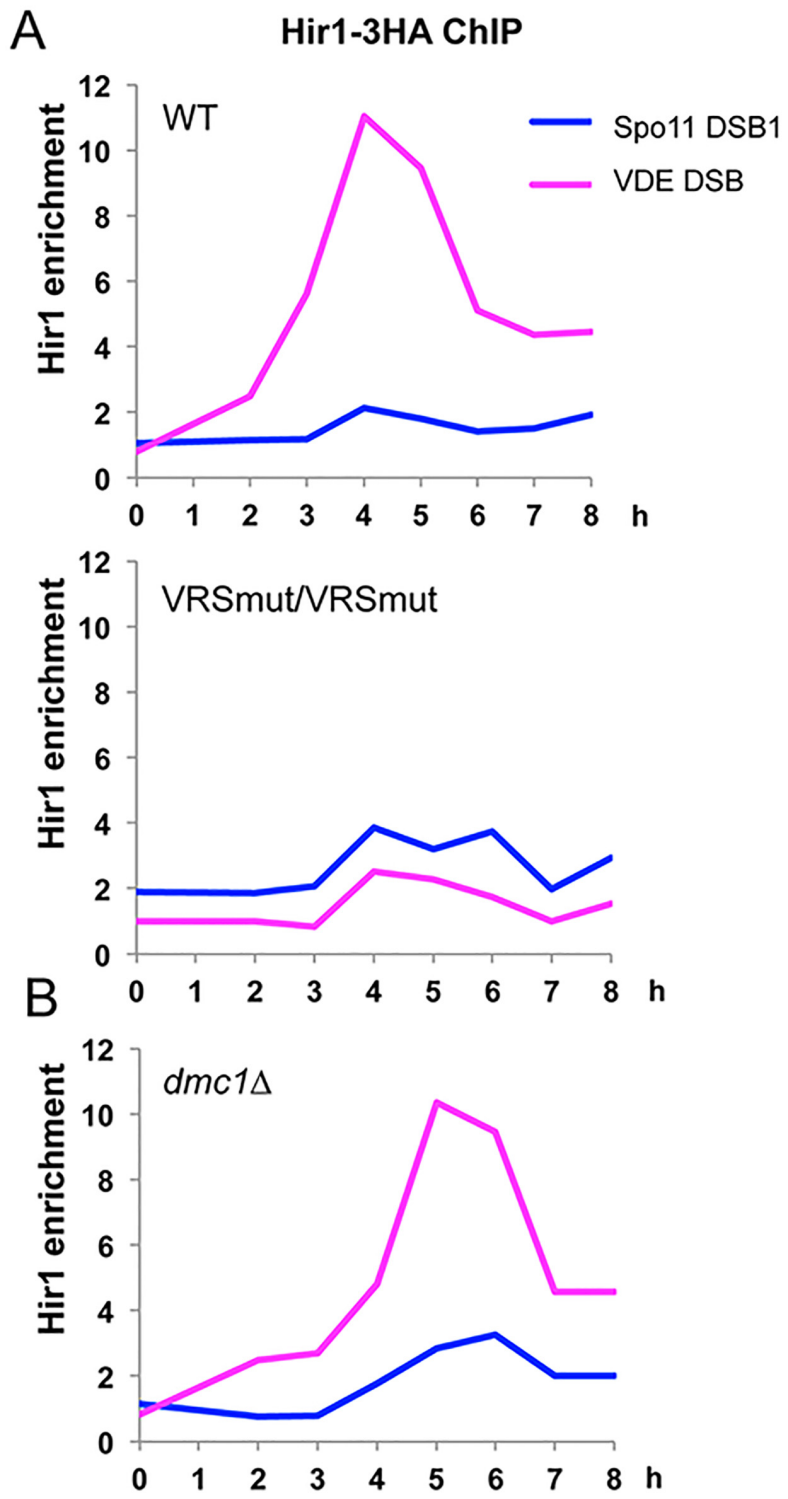
The Hir histone chaperone is recruited to meiotic DSB. (A) Hir1-3HA association with the VDE DSB site and one Spo11 DSB site (*GAT1*) relative to the negative control site. ChIP from VBD1292 (VRS::*ARE1*/VRS mut::*ARE1*) and from VBD1322 (VRS mut::*ARE1*/VRS mut::*ARE1*) meiotic time course. (B) Hir1-3HA association as in A from a *dmc1Δ* VRS::ARE1/VRS mut::ARE1 time-course (VBD1312).

CAF-1 is the major histone chaperone that promotes histone incorporation during DNA synthesis, but its absence can be compensated by the HIRA (Hir in budding yeast) histone chaperone, which promotes histone incorporation independently of DNA synthesis [34]. We thus asked if the Hir complex (composed of Hir1, Hir2, Hir3 and Hpc2 subunits [41]) is also detected at meiotic DSBs. We found that the Hir1 subunit associates with the cleaved VDE DSB site and not in a strain containing both mutated VRS sites (Fig 4A). It also associated, but to a weaker extent, to the *GAT1* Spo11 hotspot (Spo11 DSB1). Interestingly, similarly to CAF-1, Hir1 also associated with meiotic DSBs in a *dmc1Δ* mutant (Fig 4B).

This indicates that the DNA synthesis-independent Hir chaperone can associate with meiotic DSBs when there is no strand invasion, like CAF-1.

### Deletion of CAF-1 histone chaperone does not affect meiotic DSB repair or rates of crossover formation

**Fig 5:**
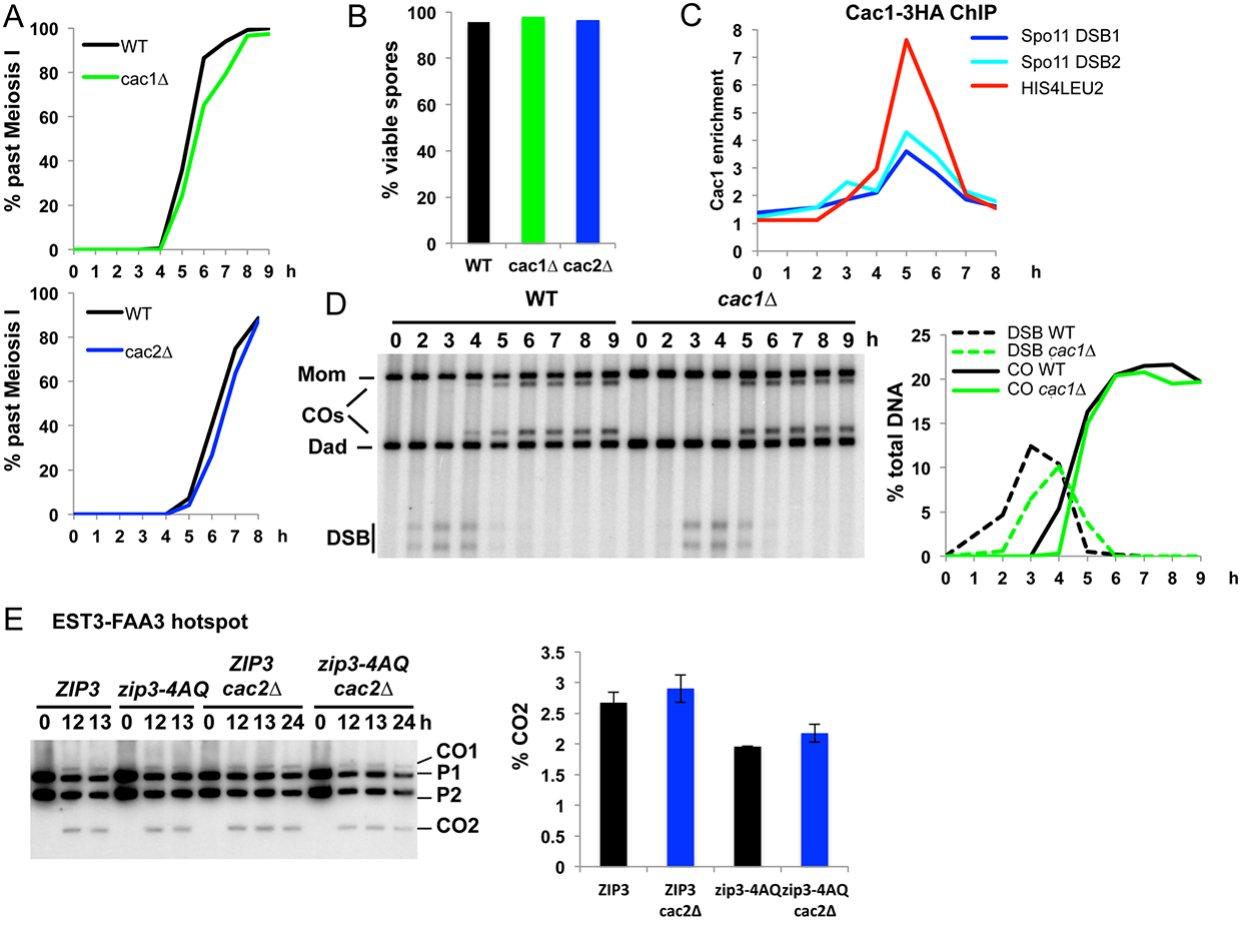
Cac1 deletion does not affect crossover levels nor spore viability. (A) Meiotic progression was followed by DAPI staining in WT (VBD1311), *cac1Δ* (VBD1341) and *cac2Δ* (VBD1262) strains. Past Meiosis I: nuclei having passed the first meiotic division. (B) Spore viability in WT (VBD1311; 155 tetrads), *cac1Δ* (VBD1341; 175 tetrads) and *cac2Δ* (VBD1262; 213 tetrads) strains. (C) Cac1-3HA association with *HIS4LEU2*, *GAT1* (DSB1) and *BUD23* (DSB2) Spo11 sites relative to negative control site. ChIP was performed in a VBD1379 meiotic time course. (D) DSB formation and CO frequency at *HIS4LEU2* in WT (VBD1311) and *cac1Δ* (VBD1341) monitored by Southern blot. The graph shows DSB and CO quantification from the same time-courses. (E) CO frequency at the *EST3-FAA3* locus in *ZIP3* (VBD1229), *zip3-4AQ *(VBD1113), *cac2Δ ZIP3* (VBD1282) and *cac2Δ zip3-4AQ* (VBD1283) monitored by Southern blot. The positions of parental bands (P1 and P2) and of the recombinant crossover products (CO1 and CO2) are indicated. The graph shows mean CO2 frequency between the 12h and 13h time-points ± standard deviation.

Next we investigated if the CAF-1 recruitment to meiotic DSBs has any impact on their repair. *cac1Δ* or *cac2Δ* cells, mutated in the large or middle CAF-1 subunits, respectively, progressed into meiotic divisions with a slight delay of less than half hour (Fig 5A) but showed wild-type spore viability (Fig 5B). In addition, *cac1Δ* cells did not show pronounced delay in meiotic S phase kinetics (S1 Fig). We monitored recombination at the well-characterized *HIS4LEU2* hotspot [3], where CAF-1 binds during meiosis (Fig 5C). *cac1Δ* strains formed DSB at normal levels, with a short delay, and produced wild-type crossover levels at the *HIS4LEU2* hotspot (Fig 5D). Similarly, genetic distances measured in two intervals on chromosome VIII were not significantly changed in the *cac1Δ* mutant (Table 1).

**Table 1:** Tetrad analysis of genetic distances and crossover interference in the *cac1Δ* mutant.

Genotype	reference interval		*CEN8-ARG4*	*ARG4-THR1*
		test interval	*ARG4-THR1*	*CEN8-ARG4*
**Wild-type**	TT+NPD	PD:NPD:TT	361:0:21	181:0:21
		cM ± SE	2.75 ± 0.58	5.2 ± 1.07
	PD	PD:NPD:TT	877:0:181	877:6:355
		cM ± SE	8.55 ± 0.58	15.79 ± 0.85
	ratio		0.32	0.33
	sig. (p)		1.3E-09	2.1E-09
	total	PD:NPD:TT	1238:0:202	1058:6:376
		cM ± SE	7.01 ± 0.46	14.31 ± 0.76
***cac1Δ***	TT+NPD	PD:NPD:TT	397:0:26	190:0:26
		cM ± SE	3.07 ± 0.58	6.02 ± 0.11
	PD	PD:NPD:TT	986:0:190	986:5:392
		cM ± SE	8.08 ± 0.54	15.26 ± 0.76
	ratio		0.38	0.39
	sig. (p)		3.1E-8	4.8E-8
	total	PD:NPD:TT	1383:0:216	1176:5:418
		cM ± SE	6.75 ± 0.43	14.01 ± 0.68

Genetic distances were compared by applying the G test to the distribution of parental ditype (PD), non-parental ditype (NPD) and tetratype (TT).

To test the hypothesis that CAF-1 nucleosome assembly may act redundantly with the ZMM proteins that protect recombination intermediates, we next examined strains with a weakened ZMM pathway, bearing a hypomorph mutation of the Zip3 ZMM protein, *zip3-4AQ*, which lowers crossover frequency without activating the Mus81 pathway [42]. *cac2Δ* combined with this mutation did not further decrease crossover frequency, suggesting that CAF-1 is not acting redundantly with ZMM to promote crossover (Fig 5E).

Next we asked if the minor non-interfering class II crossover pathway is sensitive to CAF-1 inactivation, by two different strategies.

**Fig 6:**
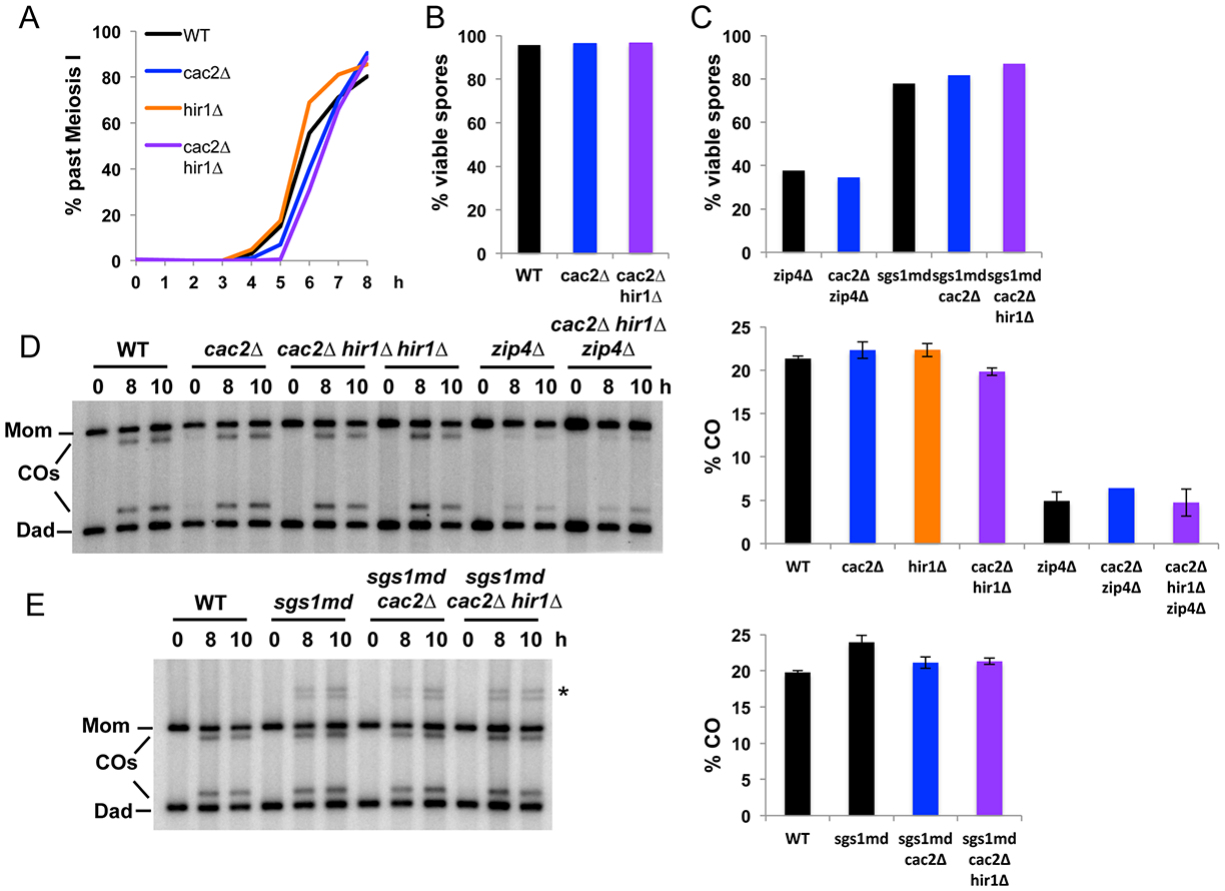
CAF-1 and Hir mutants have no CO defect in the class II crossover pathway. (A) Meiotic progression in WT (VBD1311), *hir1Δ* (VBD1310), *cac2Δ* (VBD1262) and *cac2Δ hir1Δ* (VBD1304) cells. (B) Spore viability in WT (VBD1311; 155 tetrads), *cac2Δ* (VBD1262; 213 tetrads) and *cac2Δ hir1Δ* (VBD1304; 192 tetrads) cells. (C) Spore viability in *zip4Δ* (VBD1082; 128 tetrads), *zip4Δ cac2Δ* (VBD1270; 128 tetrads), *sgs1-md* (VBD1288; 26 tetrads), *sgs1-md cac2Δ* (VBD1286; 26 tetrads) and *sgs1-md cac2Δ hir1Δ* (VBD1330; 52 tetrads) cells. (D) CO frequency at *HIS4LEU2* in WT (VBD1311), *cac2Δ* (VBD1262), *hir1Δ* (VBD1310), *hir1Δ cac2Δ* (VBD1304), *zip4Δ* (VBD1082) and *zip4Δ cac2Δ hir1Δ* (VBD1321) monitored by Southern blot at the indicated times in meiosis. The graphs show mean CO between the 8h and 10h time-points ± standard deviation. (E) CO frequency at *HIS4LEU2* in WT (VBD1311), *sgs1-md* (VBD1288), *sgs1-md cac2Δ* (VBD1286) and *sgs1-md hir1Δ cac2Δ* (VBD1330), monitored by Southern blot. The graphs show mean CO between the 8h and 10h time-points ± standard deviation except for the *cac2Δ zip4Δ* sample that was measured at only 10h (gel not shown). The asterisk indicates products of aberrant non-allelic crossovers more frequently observed in the absence of Sgs1 [65].

First we mutated *ZIP4*, one of the ZMM, which results in about 15% residual crossovers at *HIS4LEU2*, dependent on the class II Mus81 pathway [13]. In such situation, CAF-1 inactivation did not further reduce spore viability or crossover, meaning that CAF-1 is not important for the residual, class II, crossovers (Fig 6C and 6D, *zip4Δ cac2Δ* mutant). Second, we used a meiotic depletion allele of *SGS1*, *sgs1-md*, which redirects recombination events to the Mus81-dependent pathway [7]. In such mutants, neither spore viability nor crossover frequency was modified by deletion of *CAC2*, confirming that in Sgs1-deficient cells, CAF-1 does not participate in crossover formation together with Mus81 (Fig 6C and 6E).

**Fig 7:**
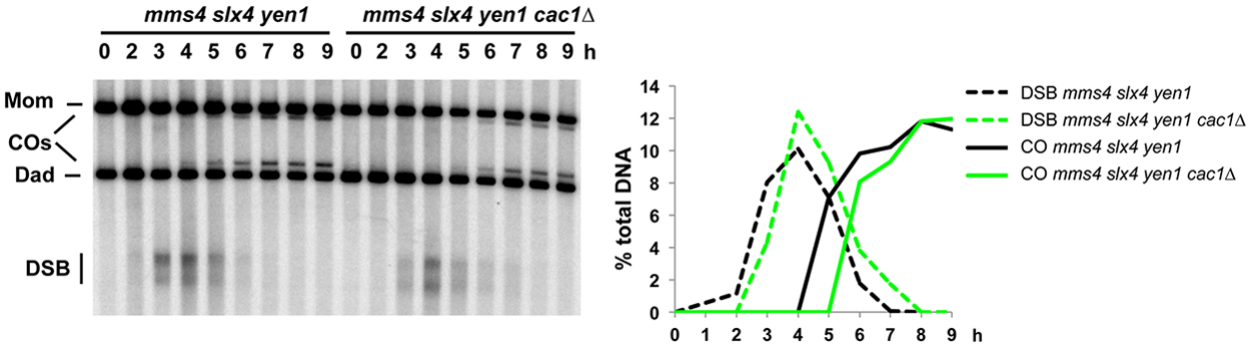
Recombination in CAF-1 mutants does not rely more of the class II crossover pathway. DSB formation and CO frequency at *HIS4LEU2* in *mms4 slx4 yen1* triple mutant (VBD1444) and in *mms4 slx4 yen1 cac1Δ* (VBD1443) monitored by Southern blot. The graph shows DSB and CO quantification from the same time-courses.

Then we asked if the absence of crossover reduction in *cac2Δ* cells could be because a weakening of the ZMM-dependent pathway could be compensated by an increased use of the class II crossover pathway. To test this hypothesis, we examined crossovers in CAF-1 mutants also deficient for the three nucleases known to promote the class II CO pathway, namely Mus81-Mms4, Slx1-Slx4 and Yen1 [6]. As previously described, the inactivation of these three nucleases reduced CO frequency at *HIS4LEU2*, by 40% [6]. However, the combined deletion of *CAC1* did not further reduce CO formation (Fig 7). We independently confirmed this observation, by showing that interference, which is promoted by the class I CO pathway, was not altered in the *cac1Δ* mutant (Table 1).

We conclude that the number of CO promoted by the class I ZMM-dependent pathway is unchanged in the absence of CAF-1.

Nucleosomes are reassembled at the VDE DSB in a single *cac1Δ* mutant, with no significant delay compared to wild-type, suggesting redundant activities for nucleosome reassembly (S2 Fig). Since both CAF-1 and Hir histone chaperones are present at meiotic DSBs, we asked if they may have redundant functions for recombination. Double *cac2Δ hir1Δ* mutants produced fully viable spores (Fig 6B). We investigated the phenotype of *hir1Δ* simple mutants and *cac2Δ hir1Δ* double mutants in both crossover pathways. Like for CAF-1 single mutant, we failed to detect any spore viability or crossover defect at the *HIS4LEU2* hotspot, both in wild-type crossover pathway or in the only presence of the Mus81 pathway (*zip4Δ* or *sgs1-md* mutant) (Fig 6C, 6D and 6E).

## Discussion

In this study, we have shown that chromatin activities that assemble nucleosomes are recruited to sites of meiotic recombination. This is the first time that histone chaperones have been found at naturally occurring DSBs, showing that reassembling histones is part of the normal meiotic program of recombination, and does not occur only at accidental lesions. Similar to what has been observed in mammalian cells, we find that CAF-1 is recruited early to DSBs, even without strand invasion intermediates and independently of its interaction with PCNA. More surprising was the finding that the other histone H3 chaperone, Hir, is timely recruited to DSBs, and also in the absence of strand invasion, uncovering a new mode of recruiting Hir to chromatin that remains to be determined. Finally, we tested the effect of these histone chaperones in meiotic recombination, but could not observe any meiotic recombination defect.

### The Hir histone chaperone is timely recruited to meiotic DSBs

For DSB repair by homologous recombination, nucleosomes are likely evicted during DSB end resection. In addition, several studies have reported a loss of histones on the donor template ([19] and this study), likely to allow for D-loop extension and the formation of joint molecules. It is thus expected that histones lost on the donor template are replaced, independently of DNA synthesis. The Hir complex is a good candidate to fulfill this role, performing DNA synthesis-independent nucleosome incorporation. However, we were surprised to detect Hir1 association not only in repair-proficient cells, but also in *dmc1Δ* cells, which do not proceed beyond end resection. In *dmc1Δ* cells, DSB accumulate with hyper-resected ends. A plausible explanation is that Hir is recruited because histones have been evicted and Hir has a tendency to bind nucleosome-free regions [34]. Thus, this recruitment of Hir in *dmc1Δ* may be due to the accumulation of DSBs, and the early Hir recruitment may not occur in wild-type cells that proceed quickly to recombinase loading and strand invasion. It is interesting to note that recently, HIRA recruitment to DSB lesions has been observed in mammalian cells upon laser-irradiation [35], although the mode of recruitment is unknown. However, this “gap-filling” function of HIRA at nucleosome-free regions is challenged by the recent discovery that after UV damage, HIRA is quickly recruited to UV lesions, depending on the CUL4A-DDB1 ubiquitin ligase. Its proposed role is to “licence” the chromatin by loading new histones and allowing transcription restart [36].

### Cac1 early recruitment is independent of PCNA interaction

Since CAF-1 preferentially reassembles nucleosomes coupled to DNA synthesis, its involvement would have been predicted to take place during the DNA synthesis step that follows strand invasion and D-loop formation. In addition, the prediction is that at this step, it should need interaction with PCNA.

However, we found that both Cac1 and Cac2 are recruited early to DSBs, independently of Cac1 interaction with PCNA. This is reminiscent to what is seen in mammals, where the large subunit of CAF-1 is recruited to laser induced DSBs, and through the recruitment of HP1, facilitates resection [30]. Both studies (Baldeyron’s and ours) thus uncovered a new mode recruitment of CAF-1 recruitment, likely preceding the step of chromatin assembly.

The early recruitment of CAF-1 does not mean that PCNA is not required later, to couple DNA synthesis with chromatin assembly on the recombination intermediates. As proposed at DSB in mammalian cells, CAF-1 may first be recruited to meiotic DSBs and then switch to an active histone chaperone to restore chromatin upon DNA synthesis and interaction with PCNA [30]. In this regard, it would be interesting to assess the extent of spreading of the Cac1-20 mutant, unable to interact with PCNA, around the VDE DSB as compared to the wild-type Cac1.

In Mammals, the p60 CAF-1 subunit is not required for early Cac1 recruitment to DSBs and HP1 recruitment to promote resection [30]. Similarly, we found that the equivalent yeast Cac2 subunit is not required for Cac1 loading. However, the whole complex seems to be recruited early, since we show that Cac2 is also fully recruited to DSBs in *dmc1Δ*.

### What could be the factors involved in recruiting Cac1 to DSBs?

In the fungus *Coprinus*, CAF-1 was found to interact with Dmc1, and it was proposed that CAF-1 might be recruited to meiotic recombination intermediates through this interaction, to promote nucleosome assembly [43]. However, our data do not fit with this model, since we observe CAF-1 recruitment in the absence of Dmc1. An alternative possibility is that it may be recruited through interaction with the related Rad51, but we have found that Cac1 is still recruited in the *dmc1Δ rad51Δ* double mutant (data not shown).

Could CAF-1 be recruited early because meiotic DSBs are programmed DSBs? In this case, Cac1 might be recruited via a recombination protein to ensure it is present at a later step when it is needed. A similar situation is seen for the Mre11 complex that is recruited to hotspots prior to meiotic DSB formation by interacting with some of the DSB proteins [44, 45]. For comparison, it would be interesting to test at a site-specific DSB induced in somatic cells if CAF-1 is also recruited in the absence of strand invasion.

In mammalian cells, complex purification associated with the large p150 CAF-1 subunit has identified the DSB repair protein, Ku, which may provide a way to recruit CAF-1 to DSBs [46]. However, Ku, which is important for non-homologous end-joining, is down-regulated in meiotic cells and does not act during meiotic recombination [47, 48]. It would therefore be informative to purify Cac1-associated complex from meiotic cells to identify its way of recruitment to sites of recombination.

### Role of CAF-1 recruitment to DSBs

In Mammals, the early recruitment of CAF-1 to DSB impacts the efficiency of resection, through the recruitment of HP1 and downstream resection factors [30]. In our case, *cac1Δ* mutants show no resection defect, as seen on Southern blot analysis, and our meiotic time-courses in *cac1Δ* do not show any delay in meiotic DSB repair (Fig 5D). Thus, the early function of CAF-1 does not seem conserved. It is worth mentioning that resection of meiotic DSB proceeds differently than that of DSB in cycling cells, in particular it is independent of Sgs1 [49]. In addition, in mammalian cells, CAF-1 recruits HP1 to DSBs, a protein that is not present in *S*. *cerevisiae*, to promote resection. The functional impact, if any, of early CAF-1 recruitment to DSB is thus different between *S*. *cerevisiae* and mammalian cells.

Several evidences have shown that CAF-1 and Hir (HIRA in mammals) can have redundant functions. In budding yeast, upon DNA DSB formation, inactivation of both CAF-1- and Hir1-mediated histone deposition is needed to observe a defect of checkpoint deactivation [21]. However, we have not detected any defect in the recombination outcome of CAF-1 mutants in combination with Hir mutant. We cannot exclude the possibility that a third component may reassemble histones on recombination intermediates. Recently, a triple mutant analysis screen has revealed that the Rdh54 translocase likely fulfills this function when both CAF-1- and Hir-dependent pathways are inactive, revealing redundant activities [50]. However, studying specifically the function of Rdh54 for chromatin dynamics may be challenging since it fulfills already known important functions for meiotic recombination [51].

Both in mitotically dividing budding and fission yeasts, deleting CAF-1 complex has no effect on DSB repair by homologous recombination [21, 52]. However, in vegetatively growing fission yeast, it was recently shown that the CAF-1 complex is important for the stability of recombination intermediates that in its absence are dismantled by Rqh1, the Sgs1 *S*. *pombe* homolog. This function requires interaction with PCNA, suggesting a role for the DNA synthesis-coupled chromatin assembly [52]. This result points to a role for CAF-1 in assembling chromatin during repair, and this assembled chromatin protecting recombination intermediates against helicases. We did not see such effect of either *CAC1/CAC2* deletion alone or double *CAC2 HIR1* deletion on meiotic recombination intermediates, as judged by the outcome of recombination. However, meiotic cells have a meiosis-specific, dedicated group of proteins to protect recombination intermediates against helicases function, the ZMM proteins [7]. ZMM may thus play the same role as CAF-1 plays in *S*. *pombe*. Our attempts to reveal any role of CAF-1 in recombination intermediates stability by studying the ZMM-independent crossover pathway were complicated by the fact that no good experimental condition exists that would bypass the requirement for ZMM and still keep anti-crossover helicase active. Indeed, in ZMM mutants, only a subset of crossovers are formed by the class II pathway, and the analysis is made complex by the fact that compensatory mechanisms are activated, that increase DSB frequency in order to compensate for the loss of crossovers [53]. On the other hand, totally bypassing the ZMM pathway requires inactivating Sgs1, the main anti-crossover helicase [7].

### Possible impact on the epigenetic landscape of histone replacement at sites of meiotic recombination

To conclude, we have shown in this paper that histone chaperones are present at meiotic recombination sites, likely to reassemble histones after recombination. Since epigenetic marks on newly synthesized histones are different than on parental ones, especially for histone H3 methylation [54], the incorporation of new histones at the sites that experienced a recombination event has the potential to modify the epigenetic profile. Meiotic recombination events being different from cell to cell, this epigenetic variation may add another layer of genome diversity produced by meiotic recombination in addition to genetic diversity.

## Materials and Methods

### Yeast strains

All yeast strains (S1 Table) are of the SK1 background [55]. Desired genotypes were obtained by direct transformation or by genetic crossing. All transformants were confirmed to have the correct construct integration by locus-specific PCR analysis.

The drug-resistance cassettes KanMX4, HphMX4 and NatMX4 were PCR-amplified from plasmids pFA6a, pAG32 and pAG25 [56, 57], respectively, with primers having 50 bp flanking homology to the targeted locus, and integrated by transformation for precise replacement of the coding sequence of the *CAC1*, *CAC2*, *HIR1*, *SPO11*, *DMC1* and *ZIP4* genes with the indicated resistance marker.

*CAC1*, *CAC2* and *HIR1* C-terminal tags were obtained by integrating a PCR-amplified 3HA-KanMX cassette [58] with flanking homology to each corresponding locus. The KanMX cassette was exchanged to NatMX or HphMX by yeast transformation and integration using the sequence homology between these selection cassettes. *sgs1-md* was generated by replacing the 50 bp upstream of *SGS1* with a KanMX-pCLB2 construct, as described [59, 60].

Integration of the 200 bp VRS or VRS mut allele into the *ARE1* target site was performed exactly as described [39].

### Meiotic time courses

For synchronous meiosis, cells were grown in SPS pre-sporulation medium and transferred to 1% potassium acetate with vigorous shaking at 30°C as previously described [42]. Progression through meiosis was monitored by scoring nuclear divisions after DAPI staining. Spore viability was measured after sporulation on solid sporulation medium for two days at 30°C.

### Tetrad analysis of recombination on chromosome VIII

For genetic distances on chromosome VIII, diploids were sporulated in liquid medium as described above, and recombination between fluorescent markers was scored after 24h in sporulation, by microscopy analysis as described [61]. Image acquisition was performed on workstations of the PICT-IBiSA Pasteur Imaging facility of Institut Curie. Two independent sets of each WT and *cac1Δ* strains sporulation experiments were combined. PD (parental ditype), NPD (non-parental ditype) and TT (tetratype) were scored to calculate the genetic distances and the interference between the adjacent *CEN8-ARG4* and *ARG4-THR1* intervals, as described in [62]. Briefly, tetrads were divided into two subclasses, those with a crossover within the test interval and those without. For these two subclasses, map distances were then calculated for each neighboring interval. The strength of interference can be estimated from the ratio of map distances calculated for the two subclasses. A ratio of significantly less than one indicates significant positive interference.

### Physical detection of DSBs and COs by Southern blotting

Cells were harvested from meiotic time courses at the indicated time points. For CO detection at *EST3-FAA3*, genomic DNA was prepared in low melting temperature agarose plugs and digested with the appropriate restriction enzyme as described [42, 63]. For DSB detection on whole chromosome III by pulsed-field gel electrophoresis, genomic DNA was prepared in low melting agarose plug and electrophoresed as described [63]. For VDE DSB and DSB and CO detection at *HIS4LEU2*, genomic DNA was extracted as described [63]. After restriction enzyme digestion and Southern blot, signal was quantified using a Phosphorimager (Typhoon, GE Healthcare) as described [64]. Restriction enzyme for genomic DNA digestion and coordinates of probes amplified by PCR were as follows: For *BUD23 and VRS*::*ARE1* DSB, *Bgl*II and probe from nt 214994 to 216445 of chr3;, for *HIS4LEU2* DSB and CO, *Xho*I and probe A [3]. The pulsed-field gel was probed with *CHA1*, at the left end of chromosome III.

### Chromatin immunoprecipitation and real-time quantitative PCR

For each time point, 10 ml of meiotic culture (2.10^8^ cells) were processed as described [22]. Cells were lyzed in 140 mM NaCl Lysis buffer plus 1mM PMSF, 50μg/mL Aprotinin and 1X Complete Mini EDTA-Free (Roche). We used 30 μl Protein G magnetic beads (New England Biolabs) and 2 μl of rabbit polyclonal anti histone H3Cter (Abcam no. 1791) or 50 μl PanMouse IgG magnetic beads (Life Technologies) and 5 μl (1 μg) of mouse monoclonal anti HA (HA.11 clone 16B12, Covance). Quantitative PCR was performed from the immunoprecipitated DNA or the whole cell extract using a 7900HT Fast Real-Time PCR System (Applied Biosystems) and SYBR Green PCR master mix (Applied Biosystems) as described [22]. Results were expressed as the % of DNA in the total input present in the immunoprecipitated sample, normalized with the negative control, which is neither a DSB site nor a Rec8 site, and is located in the promoter of *CDC39*, on chromosome III [42]. For Cac1-3HA, Cac2-3HA and Hir1-3HA ChIP, to minimize variations between experiments, we always processed a control reference sample in parallel (from VBD1115 at 4h), and cross-normalized all the data using this reference sample. S2 Table indicates primer sequences and the coordinates of amplified fragments.

## Supporting Information

S1 Table Yeast strains used in this study. (DOCX)

S2 Table Primers used for qPCR analysis. (DOCX)

S1 Fig Kinetics of meiotic replication in wild-type and *cac1Δ* mutant. Cell DNA content was measured by flow cytometry of Sytox green-stained cells. Meiotic time-courses of wild-type (VBD1311) and *cac1Δ* (VBD1341) were performed in parallel.
(TIF)

S2 Fig Histone H3 occupancy at the VDE DSB in *cac1Δ* cells. (A) Experimental system. Same as in Fig 1A. (B) VDE DSB formation upon time course of *cac1Δ* strain (VBD1398) monitored by Southern blot. DNA at the indicated times was digested with *Bgl*II restriction enzyme. The graph shows VDE DSB quantification in both the *cac1Δ* and the wild-type (VBD1386, from Fig 1A) strains for comparison. (C) Relative histone H3 occupancy in the proximity of the break, using qPCR primers located 250bp away on each side of the VDE break. ChIP of histone H3 from *cac1Δ* (VBD1398, solid lines) and from wild-type (VBD1386, from Fig 1A, dotted lines) meiotic time course. Error bars represent standard deviation from two independent ChIP experiments. Histone occupancy is measured relative to histone occupancy at 9.9 kb 3’ from the VDE break.
(TIF)
