# Supplementary material for: The CAF-1 and Hir Histone Chaperones Associate with Sites of Meiotic Double-Strand Breaks in Budding Yeast
Source: PLoS One. 2015 May 4;10(5):e0125965. doi: 10.1371/journal.pone.0125965 (PMC4418760; doi:10.1371/journal.pone.0125965)
Supplement: S1 Table — (DOCX) [file pone.0125965.s001.docx]

Table S1: diploid strains used in this study. The MATa parent is indicated first

**Strain name Genotype**

VBD1386 *a/l ho::LYS2 ura3/'' lys2/" leu2/" arg4/’’ VMA1(+)/'' YCR045C/ycr045C∆::URA3 YCR048W::VRSmut/YCR048W::VRS thr4∆::Aat*II*::LEU2/THR4 ZIP3-His6-Flag::KanMX/"*

VBD1389 *a/l ho::LYS2 ura3/'' lys2/" leu2/" arg4/’’ VMA1(+)/'' YCR045C/ycr045C∆::URA3 YCR048W::VRSmut/YCR048W::VRSmut thr4∆::Aat*II*::LEU2/THR4 ZIP3-His6-Flag::KanMX/"*

VBD1098 *a/l ho::LYS2 ura3/'' lys2/" leu2::hisG/’’ his4X/’’ arg4-nsp/’’ CAC1-3HA::KanMX/''*

VBD1101 *a/l ho::LYS2 ura3/'' lys2/" leu2::hisG/’’ his4X/’’ arg4-nsp/’’ CAC1-3HA::KanMX/'' spo11(Y135F)::NatMX/’’*

VBD1115 *a/l ho::LYS2 ura3/'' lys2/" leu2/" arg4-bgl/ arg4-nsp VMA1(+)/'' YCR048W::VRSmut/YCR048W::VRS ZIP3-His6-Flag::KanMX/" CAC1-3HA::NatMX/''*

VBD1161 *a/l ho::LYS2 ura3/'' lys2/" leu2/" arg4/’’ VMA1(+)/'' YCR048W::VRSmut/YCR048W::VRSmut ZIP3-His6-Flag::KanMX/" CAC1-3HA::NatMX/''*

VBD1211 *a/l ho::LYS2 ura3/'' lys2/" leu2/" arg4-bgl/ arg4-nsp VMA1(+)/'' YCR048W::VRSmut/YCR048W::VRS ZIP3-His6-Flag::KanMX/" CAC1-3HA::NatMX/'' spo11∆::HphMX/”*

VBD1206 *a/l ho::LYS2 ura3/'' lys2/" leu2/" arg4-bgl/ arg4-nsp VMA1(+)/'' YCR048W::VRSmut/YCR048W::VRS ZIP3-His6-Flag::KanMX/" CAC1-3HA::NatMX/'' dmc1Δ::HphMX/’’*

VBD1331 *a/l ho::LYS2 ura3/'' lys2/" leu2/" arg4-bgl/ arg4-nsp VMA1(+)/'' YCR048W::VRSmut/YCR048W::VRS ZIP3-His6-Flag::KanMX/" CAC2-3HA::NatMX/''*

VBD1349 *a/l ho::LYS2 ura3/'' lys2/" leu2/" arg4-bgl/ arg4-nsp VMA1(+)/'' YCR048W::VRSmut/YCR048W::VRS ZIP3-His6-Flag::KanMX/" CAC2-3HA::NatMX/'' dmc1Δ::HphMX/’’*

VBD1314 *a/l ho::LYS2 ura3/'' lys2/" leu2/" arg4-bgl/ arg4-nsp VMA1(+)/'' YCR048W::VRSmut/YCR048W::VRS ZIP3-His6-Flag::KanMX/" cac1-20-3HA::NatMX/''*

VBD1281 *a/l ho::LYS2 ura3/'' lys2/" leu2/" arg4-bgl/ arg4-nsp VMA1(+)/'' YCR048W::VRSmut/YCR048W::VRS ZIP3-His6-Flag::KanMX/" CAC1-3HA::NatMX/'' cac2∆::HphMX/”*

VBD1292 *a/l ho::LYS2 ura3/'' lys2/" leu2/" arg4-bgl/ arg4-nsp VMA1(+)/'' YCR048W::VRSmut/YCR048W::VRS ZIP3-His6-Flag::KanMX/" HIR1-3HA::NatMX/''*

VBD1322 *a/l ho::LYS2 ura3/'' lys2/" leu2 arg4-bgl/” VMA1(+)/'' YCR048W::VRSmut/YCR048W::VRSmut ZIP3-His6-Flag::KanMX/" HIR1-3HA::NatMX/''*

VBD1312 *a/l ho::LYS2 ura3/'' lys2/" leu2/" arg4-bgl/ arg4-nsp VMA1(+)/'' YCR048W::VRSmut/YCR048W::VRS ZIP3-His6-Flag::KanMX/" HIR1-3HA::NatMX/'' dmc1Δ::LEU2/’’*

VBD1311 *a/l ho::hisG/" leu2::hisG/’’ ura3/'' HIS4::LEU2-(BamH1; +ori)/his4-X::LEU2-(NgoMIV; +ori)-URA3*

VBD1341 *a/l ho::hisG/" leu2::hisG/’’ ura3/'' HIS4::LEU2-(BamH1; +ori)/his4-X::LEU2-(NgoMIV; +ori)-URA3 cac1Δ::HphMX/''*

VDB1262 *a/l ho::hisG/" leu2::hisG/’’ ura3/'' HIS4::LEU2-(BamH1; +ori)/his4-X::LEU2-(NgoMIV; +ori)-URA3 cac2Δ::KanMX/''*

VBD1379 *a/l ho::hisG/" leu2::hisG/’’ ura3/'' HIS4::LEU2-(BamH1; +ori)/his4-X::LEU2-(NgoMIV; +ori)-URA3 Cac1-3HA::NatMX/''*

VBD1229 *a/l ho::LYS2 ura3/'' lys2/" leu2-R/leu2-K arg4-nsp,bgl/’’ est3Δ::NatMX/EST3 FAA3/faa3Δ::HphMX ZIP3-His6-Flag::KanMX/"*

VBD1113 *a/l ho::LYS2 ura3/'' lys2/" leu2-R/leu2-R arg4-nsp,bgl/’’ est3Δ::NatMX/EST3 FAA3/faa3Δ::HphMX zip34AHis6-Flag3::KanMX/"*

VBD1282 *a/l ho::LYS2 ura3/'' lys2/" leu2/" arg4-nsp,bgl/’’ est3Δ::NatMX/EST3 FAA3/faa3Δ::HphMXZIP3-His6-Flag::KanMX/" cac2Δ::KanMX/''*

VBD1283 *a/l ho::LYS2 ura3/'' lys2/" leu2/" arg4-nsp,bgl/’’ est3Δ::NatMX/EST3 FAA3/faa3Δ::HphMX zip34AHis6-Flag3::KanMX/" cac2Δ::KanMX/''*

VBD1310 *a/l ho::hisG/" leu2::hisG/’’ ura3/'' HIS4::LEU2-(BamH1; +ori)/his4-X::LEU2-(NgoMIV; +ori)-URA3 hir1Δ::NatMX/''*

VBD1304 *a/l ho::hisG/" leu2::hisG/’’ ura3/'' HIS4::LEU2-(BamH1; +ori)/his4-X::LEU2-(NgoMIV; +ori)-URA3 hir1Δ::NatMX/'' cac2Δ::KanMX/''*

VBD1082 *a/l ho::hisG/" leu2::hisG/’’ ura3/'' HIS4::LEU2-(BamH1; +ori)/his4-X::LEU2-(NgoMIV; +ori)-URA3 zip4Δ::HphMX/''*

VBD1270 *a/l ho::hisG/" leu2::hisG/’’ ura3/'' HIS4::LEU2-(BamH1; +ori)/his4-X::LEU2-(NgoMIV; +ori)-URA3 zip4Δ::HphMX/'' cac2Δ::KanMX/''*

VBD1288 *a/l ho::hisG/" leu2::hisG/’’ ura3/'' HIS4::LEU2-(BamH1; +ori)/his4-X::LEU2-(NgoMIV; +ori)-URA3 pCLB2-HA3-SGS1::KanMX*

VBD1286 *a/l ho::hisG/" leu2::hisG/’’ ura3/'' HIS4::LEU2-(BamH1; +ori)/his4-X::LEU2-(NgoMIV; +ori)-URA3 pCLB2-HA3-SGS1::KanMX cac2Δ::HphMX/''*

VBD1330 *a/l ho::hisG/" leu2::hisG/’’ ura3/'' HIS4::LEU2-(BamH1; +ori)/his4-X::LEU2-(NgoMIV; +ori)-URA3 pCLB2-HA3-SGS1::KanMX cac2Δ::HphMX/'' hir1Δ::NatMX/''*

VBD1321 *a/l ho::hisG/" leu2::hisG/’’ ura3/'' HIS4::LEU2-(BamH1; +ori)/his4-X::LEU2-(NgoMIV; +ori)-URA3 zip4Δ::HphMX/'' cac2Δ::KanMX/'' hir1Δ::NatMX/''*

VBD1444 *a/l ho::hisG/" leu2::hisG/’’ ura3/'' HIS4::LEU2-(BamH1; +ori)/his4-X::LEU2-(NgoMIV; +ori)-URA3 pCLB2-HA3-MMS4::KanMX slx4Δ::HphMX/'' yen1Δ::HphMX/''*

VBD1443 *a/l ho::hisG/" leu2::hisG/’’ ura3/'' HIS4::LEU2-(BamH1; +ori)/his4-X::LEU2-(NgoMIV; +ori)-URA3 pCLB2-HA3-MMS4::KanMX slx4Δ::HphMX/'' yen1Δ::HphMX/'' cac1∆::NatMX/"*

VBD1531 *a/l ho::LYS2 leu2::hisG/’’ trp1::hisG/” ura3/'' CEN8/CEN8::tdTomato-LEU2 ARG4/ARG4::GFP*-URA3 THR1::m-Cerulean-TRP1/THR1^a^*

VBD1532 *a/l ho::LYS2 leu2::hisG/’’ trp1::hisG/” ura3/'' CEN8/CEN8::tdTomato-LEU2 ARG4/ARG4:: GFP*-URA3 THR1::m-Cerulean-TRP1/THR1 cac1∆ ::HphMX/"*

^a^: haploid parents are from Thacker D. et al (2011) Genetics 189:423-439.
