# Supplementary material for: The CAF-1 and Hir Histone Chaperones Associate with Sites of Meiotic Double-Strand Breaks in Budding Yeast
Source: PLoS One. 2015 May 4;10(5):e0125965. doi: 10.1371/journal.pone.0125965 (PMC4418760; doi:10.1371/journal.pone.0125965)
Supplement: S2 Table — (DOCX) [file pone.0125965.s002.docx]

**Table S2: Primers used for qPCR analysis**

| Name | Sequence | chr | Coordinates |
| --- | --- | --- | --- |
| - 20.35 kb from VDE DSB | F: 5’ TCGCAAGAACTTCAGGTGGAA3’  R: 5’ GGCTGAAAGTACCACGTTAGAAACT3’ | 3 | 192949 to 193016 |
| - 10.15 kb from VDE DSB | F: 5’ CCATCGTGTTCATGGATCCTT3’  R: 5’ ATTTGAACCGAACCGAATGC3’ | 3 | 203149 to 203209 |
| - 5.36 kb from VDE DSB | F: 5’GTCACAACCACAGCTAACCTCATATT3’  R: 5’ CATACCCTTCGGGAGAAAAGAA3’ | 3 | 207928 to 207992 |
| - 1.95 kb from VDE DSB | F: 5’ CGGCTGCAGATTCAAAAGC3’  R: 5’ AGCATATCCAGGCGAAGATGA3’ | 3 | 211405 to 211460 |
| - 0.25 kb from VDE DSB | F: 3’ GCAGCAATTTCTTCATGTTCTGTT3’  R: 5’ TGATGCGCGACGTTCTTG3’ | 3 | 213047 to 213121 |
| 0.04 kb from VDE DSB | F: 5’ GAGAAGGTGTGCGCCATCA3’  R: 5’ TGTGCCGTGACCATCATGA3’ | 3 | In the VRS sequence inserted at 213129 |
| 0.25 kb from VDE DSB | F: 5’TTGCGTGGCGGAGTTGA3’  R: 5’TCCACCAGTCGCCGTAGAA3’ | 3 | 213362 to 213399 |
| 2.23 kb from VDE DSB | F: 5’TCTGCACGAGTCCGCAAAC3’  R: 5’CCGCGACACCTTCCAAAA3’ | 3 | 215320 to 215378 |
| 4.97 kb from VDE DSB | F: 5’ TTGCCGATGCTGTAAACAATG3’  R: 5’ GGCAAAACATCCTTTTCAAATGA3’ | 3 | 218060 to 218126 |
| 9.9 kb from VDE DSB | F: 5’ GCGACCGTCCTCGTCAATA3’  R: 5’ TGCTGCCATTGATCTGAACAA3’ | 3 | 222995 to 223055 |
| 20.24 kb from VDE DSB | F: 5’ CTGCATTTCCGTCGTAAGCA3’  R: 5’ GCCACAAAATCAGCGAAAATGT3’ | 3 | 233338 to 233399 |
| VRS mut | F: 5’ CTGACGCCATTATCTATTCTGGC3’  R: 5’ CAAGACTTCTGCCATTTCATTACCT3’ | 3 | In the VRSmut sequence inserted at 213129 |
| Spo11 DSB1 : *GAT1* | F: 5’ CCCGGCCCCGGTATTA3’  R: 5’ GAAGGGCGGAAAAGCAAAG3’ | 6 | 95982 to 95948 |
| Spo11 DSB2 : *BUD23* | F: 5’GGCACCACCGGAGATTTTC3’  R: 5’ TGGTCGAACCCGTGTACTTG3’ | 3 | 211467 to 211525 |
| Neg. control site | F: 5’ TTTTTTCGGTGGCAACCAA3’  R: 5’ CACCTTTGCCCCTGTTTATCTT3’ | 3 | 279988 to 280029 |
| HIS4LEU2: *RRP7* | F: 5’ ATTGAAGACATTAGCGCCATGA3’  R: 5’ TGAAATGCAACGAAGCTTCCT3’ | 3 | 65541 to 65460 |
