# Supplementary figures and images for: The CAF-1 and Hir Histone Chaperones Associate with Sites of Meiotic Double-Strand Breaks in Budding Yeast
Source: PLoS One. 2015 May 4;10(5):e0125965. doi: 10.1371/journal.pone.0125965 (PMC4418760; doi:10.1371/journal.pone.0125965)

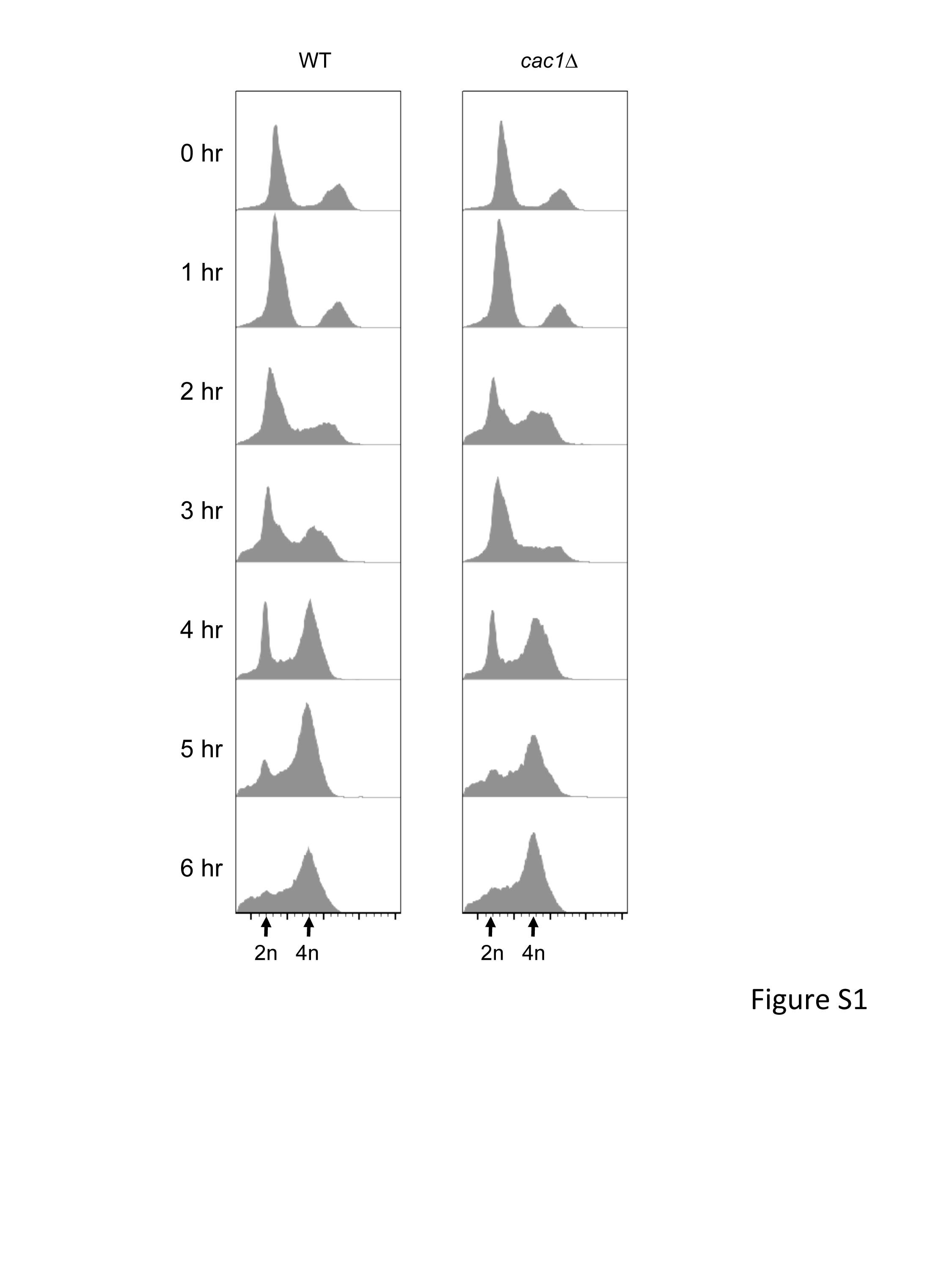

Supplement: S1 Fig — Cell DNA content was measured by flow cytometry of Sytox green-stained cells. Meiotic time-courses of wild-type (VBD1311) and cac1Δ (VBD1341) were performed in parallel. (TIF) [file pone.0125965.s003.tif]

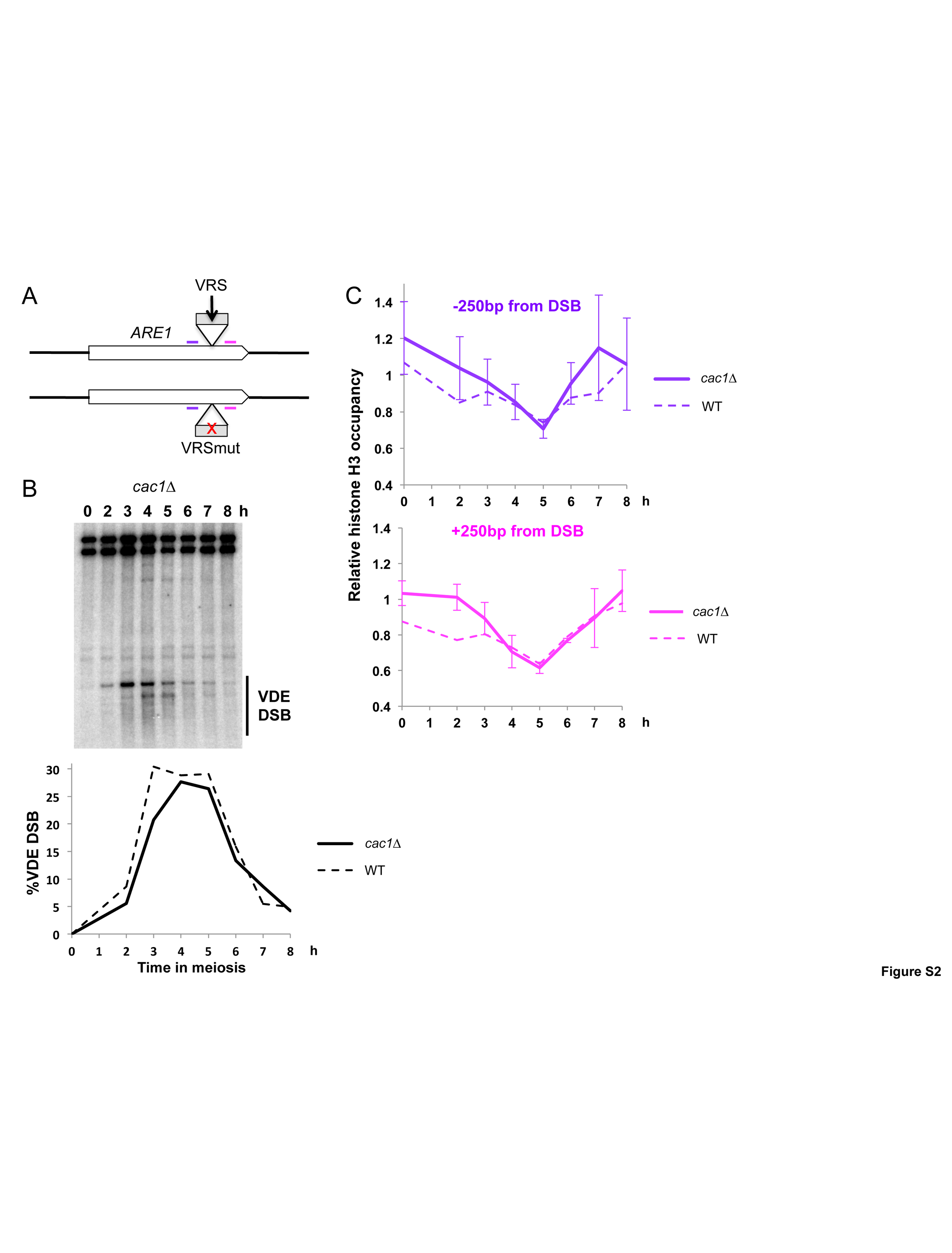

Supplement: S2 Fig — (A) Experimental system. Same as in Fig 1A. (B) VDE DSB formation upon time course of cac1Δ strain (VBD1398) monitored by Southern blot. DNA at the indicated times was digested with BglII restriction enzyme. The graph shows VDE DSB quantification in both the cac1Δ and the wild-type (VBD1386, from Fig 1A) strains for comparison. (C) Relative histone H3 occupancy in the proximity of the break, using qPCR primers located 250bp away on each side of the VDE break. ChIP of histone H3 from cac1Δ (VBD1398, solid lines) and from wild-type (VBD1386, from Fig 1A, dotted lines) meiotic time course. Error bars represent standard deviation from two independent ChIP experiments. Histone occupancy is measured relative to histone occupancy at 9.9 kb 3’ from the VDE break. (TIF) [file pone.0125965.s004.tif]
